# The Effect of Inner Engineering Online (IEO) Program on Reducing Stress for Information Technology Professionals: A Randomized Control Study

**DOI:** 10.1155/2022/9001828

**Published:** 2022-01-04

**Authors:** P. Upadhyay, T. F. H. Chang, S. Hariri, S. Rallabandi, Santha Yathavakilla, V. Novack, B. Subramaniam

**Affiliations:** ^1^Sadhguru Center for a Conscious Planet, Department of Anesthesia, Critical Care & Pain Medicine, Beth Israel Deaconess Medical Center, Boston, MA, USA; ^2^School of Management and Labor Relation, Rutgers University, New Brunswick, NJ, USA; ^3^Harvard Medical School, Boston, MA, USA; ^4^Isha Institute of Inner Sciences, TN, India; ^5^Clinical Research Center, Soroka University Medical Center, Beer-Sheva, Israel

## Abstract

In recent years, mindfulness-based interventions (MBIs) are rapidly growing in the workplace. Several meta-analyses conclude that overall MBIs have a moderate effect of alleviating deficit-based experiences, such as burnout and stress, but a small to no effect of promoting asset-based experiences, such as positive affect and well-being. While workplace MBIs vary greatly in their content, format, and duration, the dominant format is still face to face in a group setting, which limits scalability. Our study introduces an emerging workplace intervention called Inner Engineering Online (IEO) and evaluates its effect on reducing stress, burnout, depression, and anxiety and increasing mindfulness and joy. Drawing on the classical yogic science, IEO is a comprehensive web-based multicomponent intervention that utilizes dialectic discourse, meditation, and yogic practices designed to improve physical, mental, and emotional health. Utilizing a randomized active control cross-over experimental design with a sample of 71 employees of an Information Technology company, we tested our hypothesis that IEO training and regular daily yogic practice are likely to lower the stress levels, prevent burnout, and alleviate anxiety and depression, while at the same time promotes positive affect for employees. The results show that IEO program significantly reduces stress only among those who adhere to recommended daily yogic practices. The study is limited by its small sample size. Future research using a large sample is recommended to reexamine the effect of IEO training on occupational health. This trial is registered with NCT04126564.

## 1. Introduction

Burnout, stress, and mental ill-health and their adverse effects on individuals, organizations, and society have been persistent and growing among employees and employers across various occupations globally [1]. Additionally, burnout incurs organizational costs resulting from decreased productivity, high employee turnover [2], and sickness absenteeism [3]. In the United States, workplace stressors result in 120,000 deaths each year [4]. When neglected, chronic workplace stress often leads to several health problems, such as cardiovascular diseases, mental disorders, musculoskeletal pain, fatigue, and insomnia [5]. In 2008, the total healthcare cost associated with workplace stressors represented 5 to 8% of the national healthcare spending with an expenditure of $190 billion a year [4].

Given this harmful ripple effect of burnout and stress, health promotion targeting burnout and stress has become imperative at workplace. In recent years, mindfulness-based interventions (MBIs) or mindfulness-based programs (MBPs) in the workplace are growing exponentially and showing encouraging results [1]. In 2018, about 60% mid- to large-sized US companies reported offering mindfulness, yoga, or meditation courses to their employees [6].

The current explosion of interest was sparked by Jon Kabat-Zinn [7], who introduced mindfulness meditation into the field of behavioral medicine and medical research four decades ago. To date, MBSR remains the gold standard of intervention in clinical and nonclinical settings and also spurs the growth of numerous offshoot modified programs for application in a variety of contexts, including occupational and organizational contexts [1, 6, 8].

Multiple studies on workplace MBIs have concluded that overall MBIs show encouraging evidence in alleviating undesirable “deficit-based” mental health outcomes, such as stress, anxiety, and depression but present less conclusive or small effect on “asset-based” well-being outcomes, such as positive emotion and life satisfaction [1]. Among the MBIs, MBSR has demonstrated strong evidence in reducing stress in healthy adult population [8]. The progress of MBIs in occupational and organizational contexts brings challenges as well as opportunities. The present study aims to contribute to the existing literature of MBIs by tackling some of these challenges and responding to some of the opportunities.

The workplace mindfulness-based interventions (MBIs) or programs (MBPs) are highly heterogeneous in intervention content, dose, and mode. Vonderlin et al. [6] observed that several curricula are idiosyncratic, which “couple mindfulness elements with other training, such as emotion regulation, compassion, or physical exercise to produce beneficial synergistic effects” (p. 1581). Modified MBIs often do not disclose what principles and methods underlie the modified program content and the qualification of the program designers and instructors. Furthermore, the scientific foundations of modified programs are not well understood, which is likely to affect the effectiveness of the programs and lead to variation of effects [8]. The modified doses include shortened classes and meditations, ranging from 10 min self-guided meditations 5 days a week with no classes to 42 hr class time over 8 weeks, with 25 min daily practice [8]. Vonderlin et al. [6] note that, on average, the programs involved 16.9 hours of attendance and were offered over a period of 1 to 16 weeks, with an average time span of 7.5 weeks.

Next, flexible delivery methods have been examined, and Vonderlin et al. [6] observed that 79% of the programs are delivered in-person, followed by online programs (13%), combinations of online and in-class elements (7%), or via audio records (1%). A recent review of online MBSR in nonwork settings finds equivalent effects of online to those of face-to-face class-based training [1]. However, flexible delivery modes, such as online or app, have been underinvestigated in the work settings. This presents an opportunity for researchers to examine the effectiveness of online or app-based programs.

Overall, workplace MBIs demonstrate moderate effects on “deficit-based” outcomes such as stress, anxiety, and distress but are less conclusive for depression and burnout [1, 8]. The effects of MBIs are small on “asset-based” outcomes, such as health, job performance, compassion and empathy, mindfulness, and well-being, while no effect on emotional regulation [1, 8]. Follow-up data for a period up to 12 weeks after postmeasurement were reported in 18 studies (34%), with a mean time lag of 9.11 weeks [6]. Therefore, there is an opportunity to explore other underresearched science-based yogic methodologies for workplace applications that offer shorter duration, flexible online self-paced format, and scalability. Additionally, a need to move beyond MBIs developed in clinical setting and to explore other established methodologies or those designed specifically for the workplace has also been identified [1].

With this study, we seek to contribute to the current understanding of the effectiveness of body and mind interventions in the workplace by introducing a yogic methodology called “Inner Engineering Online” (IEO). Drawing on the classical yogic sciences, this program that originated in India has increased demand in the general population worldwide as well as in the corporate context in North America. IEO has been piloted in Fortunate 500 and technology companies. IEO expands the current repertoire of MBIs in the workplace by offering multilinguistic, web-based, self-paced, and comprehensive features.

Second, our study expands the occupational samples of MBIs by including the Information Technology professionals, a fast growing but understudied profession in workplace MBIs research. Consequently, by surveying the Information Technology professionals, the study also adds diversity (more men) to the current pool of workplace MBIs research participants (predominately women). The work culture in technology companies is demanding and highly stressful, often requiring employees to work long hours with irregular schedules to meet the targets [9, 10]. Entrepreneurs in technology startups often work in competitive, chaotic, and unpredictable environments that test their adaptive and innovative abilities with little room for errors. Working in such demanding environments can lead to high-stress levels, sleep disturbances, and social isolation, depleting the innate coping mechanisms to handle stress [11]. Besides, they are often underappreciated for meeting these work expectations; predisposing individuals to low self-esteem and burnout [11]. Previous studies have shown a positive association between work-related stress and poor health outcomes among the IT industry employees [12, 13]. Lastly, our study answers the call for using an active control group in meditation and yoga studies, which is a more robust research design [14, 15].

The evidence so far suggests that workplace MBIs have a moderate effect on these “deficit-based” dysfunctional outcomes such as stress, anxiety, and distress, but the evidence is less conclusive for depression and burnout [1, 8]. The aim of MBIs is to enhance mindfulness, which in turn ameliorates dysfunctional symptoms such as stress, anxiety, and depression [1, 8]. Although there are external stressors or stimuli for anxiety, one's own thoughts, emotion, and physiology are also significant sources of internal stressors and stimuli [16]. The intellectual self-inquiry aspect of IEO is to recognize both external and internal stressors, accept their inevitability at the present moment, bring a sense of curiosity to investigate their causes, and finally unroot the cause of stress by not identifying with one's own thoughts, emotions, or bodily sensations [17]. The guided meditations and physical and sound yoga aspects of IEO further assist in alleviating mental distress by improving physiological and chemical functioning [18]. Thus, we hypothesize that the synergistic effect of IEO's multicomponents of didactic inquiry of human experiences, Upa Yoga, and meditations will produce the following effects:

  Hypothesis 1: the practice of IEO intervention will result in higher levels of mindfulness and joy
  Hypothesis 2: the practice of IEO intervention will lower levels of stress, anxiety, depression, and burnout

## 2. Method

### 2.1. Participants and Procedure

Participants were recruited from a midsize Information Technology company located in the United States. The opportunity to enroll in IEO and participate in the study was offered to employees through a company-wide awareness drive. This is similar to the common recruitment method of MBI studies in which participants were mostly self-selected into the study in response to invitation campaigns [8]. Eighty-two employees expressed interest. After being screened for eligibility (aged 18 years or older, proficiency in English, and US residency), 71 employees were eligible and enrolled in the study by signing electronic consent forms. These participants completed their baseline surveys and were randomized based on a sequence of computer-generated random numbers into the two study groups: the intervention and the active control group.  Table 1 describes the sociodemographic and medical characteristics of these participants at baseline.

### 2.2. The Inner Engineering Online (IEO): The Intervention

Inner Engineering Online is a 4-week self-paced multicomponent program available online and via an app in ten languages. The program was created in 2011 by Sadhguru Jaggi Vasudev, a mystic, yogi, who founded the Isha Foundation, through which the IEO program is offered. Based on the distilled essence of yogic science, IEO consists of comprehensive methods that include conducting intellectual inquiry (resulting in cognitive reappraisal), generating positive emotions, learning Upa Yoga (preparatory Hatha Yoga involving body movement and breathwork), and activation of inner energy (sound and postural yoga).

IEO's first component is the seven online lessons (90 minutes per session) employing logic-based self-inquiry and investigation of everyday human experiences and accompanied by humorous wisdom-based stories. These didactic sessions explore Inner Engineering principles that participants are encouraged to review multiple times daily mentally. By gaining the ability to reappraise one's internal mental and physiological processes and external situations and social relations, one encounters reduced automatic reactivity to internal and external stressors.

The second component of IEO is learning and practicing a system of “Upa Yoga” (“pre” or “sub” yoga), introductory practices as part of the classical Hatha Yoga. No previous yoga or meditation experience is required, nor is physical agility. Participants learn six Upa Yoga practices that activate the joints, muscles, and energy systems and stimulate the parasympathetic nervous system. Beyond awareness of breathing as part of mindfulness meditation, Upa Yoga includes volitional control of breath (pranayama). Recent studies show that volitional control and awareness of breathing activate overlapping but distinct regions of neural network [19] and that the rhythm of breathing creates electrical activity in the brain that enhances emotional judgement and memory recall [20].

IEO also includes meditative practices that involve sound (sound yoga). Studies indicate that listening to the “AUM” sound generates emotional empathy [21] and chanting these three sounds brings physiological relaxation and mental alertness [22]. Combining cognitive reappraisal with the yoga of sound (AUM chanting) produce synergistic effects in relaxation and mental alertness and induces positive emotion such as joy. The at-home practice of Upa Yoga, sitting quietly and reviewing IE principles, and AUM chanting takes about 30 minutes daily.

The third component of IEO is guided meditation, awareness questions, and reflective writing at the end of each online lesson. The guided meditations incorporate visualization, body, and breathwork. The awareness questions and reflective writing provide participants an opportunity to contemplate on lessons learned, apply these insights to daily life examples, and deepen awareness.

Preliminary pilot results suggest that IEO enhances employee well-being (energy, joy, mindfulness, wholeness within oneself, and connection with colleagues) and positive organizational behaviors (meaningful work, psychological capital, and work engagement) [23]. Peterson et al. [24] found that completion of IEO when added with learning and practicing an additional 21-minute *Shambhavi* practice reduced perceived stress and general well-being. To our knowledge, this study is the first RCT that builds on this emerging line of research and used this comprehensive, low-cost, short-duration, multilinguistic, web-based, self-paced, and globally scalable body and mind intervention in an occupational setting.

For ease of understanding, the study has been described as phases. Following consenting and enrollment, baseline characteristics were measured (Time point 1). This phase is described as the *Enrollment Phase* in the consort diagram. Upon completion of their baseline surveys, participants were randomized based on a sequence of computer-generated random numbers into two groups: the intervention and the control group (also referred to as Active comparator group). Blinding and allocation concealment was not performed in this study. This phase concluded by taking measurement at the end of the 4th week (Time 2) wherein the intervention group finished receiving the IEO intervention while the control group was instructed to read a book of their choice for 30 minutes daily. This has been described as *Study Phase I* in consort diagram.

In the next phase of our study, there was a 4-week cross-over period when the intervention group performed no prescribed activity while the control group crossed over to receive IEO intervention. Participants took the third measurement at the end of the cross-over period (Time point 3). This phase was described as *Study Phase II*.

**Figure 1:**
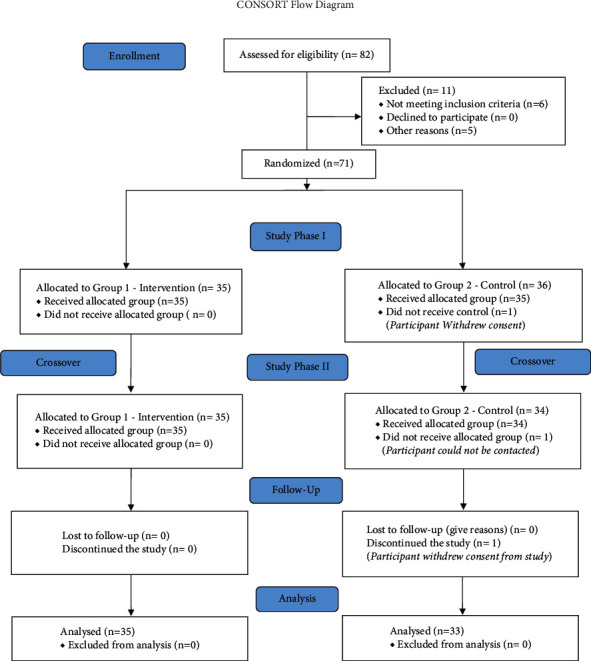
CONSORT chart.

In the final phase, a 4-week follow-up (Time point 4) was conducted. This phase is also known as the *Follow-Up phase*. The total length of the study was 12 weeks. The study protocol was approved by the lead author's institution.  Figure 1 details the various study stages.

### 2.3. Outcome Measures

1. *Mindfulness*. Mindfulness was measured using the Brown and Ryan's (2003) 15-item Mindful Attention Awareness Scale (MAAS). Each item is coded 1 “never” to 5 “all of the time.” The MAAS is one of the most commonly used mindfulness scales in the occupational setting and in general population [1]. This measure had strong reliability across study samples with Cronbach's alpha *α* = 0.92 at baseline.
2. *Stress*. Perceived stress was measured by the 10-item Perceived Stress Scale (PSS) [25]. Each item was coded as 0 “never” to 4 “very often.” The PSS score ranges from 0 to 40. The PSS is the most common measure used in stress studies with well-established reliability and validity. It is also brief and easy to administer online. The reliability of PSS is consistently high across four points of measurement with *α* = 0.82 at baseline.
3. *Anxiety*. Anxiety was measured using PROMIS-Anxiety short form v1.0 scale consisting of 7 items. Each item was coded as 1 “never” to 5 “always.” The PROMIS-Anxiety item banks assess self-reported fear (fearfulness, panic), anxious misery (worry, dread), hyperarousal (tension, nervousness, restlessness), and somatic symptoms related to arousal (racing heart, dizziness). The anxiety measures are universal rather than disease-specific and assess anxiety over the past seven days. This measure also had strong reliability across study measurements with *α* = 0.88 at baseline.
4. *Depression*. Depression was measured by the Center for Epidemiological Studies-Depression (CES-D) 20-item scale [26]. Each item was coded 1 “rarely or none of the time (less than 1 day)” to 4 “most or all of the time (5–7 days).” The CES-D scale has high reliability and validity and has remained a dominant measure for depression in community population. This measure had strong reliability across study measurement with *α* = 0.81 at baseline.
5. *Burnout*. The 16-item Maslach Burnout Inventory for General Survey (MBI-GS) [27] was used to measure burnout. The MBI-GS defines burnout as a crisis in one's relation with work and consists of three subscales: Exhaustion, Cynicism, and Professional Efficiency. The Exhaustion subscale measures both emotional and physical fatigue—one's feeling of being overextended and exhausted by one's work. Cynicism measures indifference or a distant attitude toward work. Professional Efficacy encompasses both social and nonsocial aspect of occupational accomplishments [27]. The MBI scale has high reliability across four points of measure throughout the study. Cronbach's alpha for the baseline is *α* = 0.82.
6. *Joy*. Joy was measured using the 6-item Dispositional Positive Emotional Scale—Joy (DPES-Joy) subscale [28]. Each item was coded from 1 “strongly disagree” to 7 “strongly agree.” The scale measures a dispositional tendency to feel joy in life. This measure had strong reliability across study measurements with *α* = 0.87 at baseline.

### 2.4. Statistical Analysis

The study was a feasibility trial with a minimum enrollment goal of 60 participants. Despite best efforts, we were able to enroll 71 participants in the study resulting in a small sample size in both the intervention group and the control group. The assumption of normality of sample data distribution was assessed using the Shapiro–Wilk test. The tests indicated skewed distributions of sample data; therefore, nonparametric Mann–Whitney U test was conducted to determine if IEO made a significant difference in outcomes between the intervention and control groups. Wilcoxon signed-rank test was conducted to examine the changes postintervention and during follow-up that participants experienced within their group. The median was used to indicate the central tendency of the outcomes. Categorical data were presented as frequencies and proportions and assessed using a chi-square or Fisher's exact test. IBM SPSS 25 (New York, NY, USA) was used for the analysis and two-sided *p* values were reported with a significance level at *p* < 0.05.

## 3. Results

Table 1 presents the demographic and medical conditions of participants at the baseline. Around 47% of participants in each group were males with mean ages ranging from 29 to 49, and more than half of each group had higher than a Bachelor's degree. Both the groups are comparable in their demographic profile and their baseline scores for health conditions and habits. Stress and other outcome measures showed no significant difference at study initiation. About 26.8% of participants in the intervention group and 22.9% in the control group reported prior meditation experience.

Table 2 presents the nonparametric Mann–Whitney *U* test results of intergroup comparison throughout the study duration. The results show that there was no significant difference between the intervention group and the control group in terms of median changes in stress (−3.0 *vs.* −1.5, *p* value = 0.19), anxiety (−3.0 *vs.* −2.0, *p* value = 0.3), depression (−1.0 *vs.* −1.5, *p* value = 0.79), burnout (Exhaustion subscale: (−1.0 *vs.* −1.0, *p* value = 0.81); Cynicism subscale: (−0.3 *vs.* −0.2, *p* value = 0.67); Professional Efficiency subscale: (0.5 *vs.* 0.0, *p* value = 0.55)), mindfulness (0.3 *vs* 0.4, *p* value = 0.93), and joy (0.3 *vs* 0.1, *p* value = 0.22) from baseline (T1) to Week 4 (T2). Similarly, there was no significant change in median scores of stress, burnout, depression, mindfulness, and joy between the intervention group and the control group from Week 4 (T2) to Week 8 (T3) (refer to Table 2 for scores). However, the control group showed a significant reduction in anxiety scores compared to the intervention group during the cross-over period supporting the theory that workplace MBIs demonstrate moderate effects on “deficit-based” outcomes such as stress and anxiety [1, 8] ((Phase I scores: −3.0 *vs.* −2.0, *p* value = 0.3) *vs.* (Phase II scores: 0.0 *vs.* −0.5, *p* value = 0.03)).

By taking into account the extent to which employees follow the recommended home practices routinely, we found a significant effect of IEO on the intervention group compared to the control group. Compliance was defined as 3 or more days of activity each week for at least 2 weeks out of the 4-week intervention period. Twelve (35.30%) of the intervention group participants and 23 (65.72%) of the control group participants showed sufficient compliance. We conducted further analysis to see if demographic and baseline conditions contribute to different levels of compliance but found no evidence of significant systemic differences.

**Figure 2:**
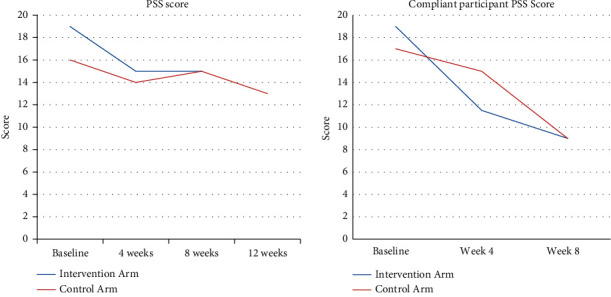
Time trend comparison between study participants.

Participants in the intervention group (−6.5 (IQR: −9.5, −1.5)) who practiced as instructed experienced a significant decline in stress compared to the control group (−2.0 (IQR: −4.0, −1.0)) between the baseline (T1) and the end of 4-week intervention (T2); (*p* value = 0.018) (see Figure 2). Once the control group participants were crossed over to receive the intervention, their stress scores also significantly declined compared to their own baseline levels ((−1.5 (IQR −4.0, −2.3)) vs. (−7 (IQR −5, −2)); *p* value = 0.085). This decline was synonymous with the decline noticed in intervention group participants during active intervention phase.

Table 3 reports the within-group, nonparametric, pairwise test (Wilcoxon signed-rank test) results. The intervention group experienced a significant decline in stress (−3.0 (IQR −8.0, 0.0), *p* value = 0.01), anxiety (−3.0 (IQR −7.0,0.0), *p* value = 0.001), and depression (−1.0 (IQR −5.0, 0.0), *p* value = 0.02) with a significant rise in both mindfulness (0.3 (IQR −0.1,0.9), *p* value = 0.01) and joy (0.3 (IQR 0.0, 0.8), *p* value = 0.03) between the baseline (T1) and the end of 4-week intervention (T2) measures. Similarly, the control group experienced a significant decline in stress (−1.5 (IQR −4.0, 1.0), *p* value = 0.04) and anxiety (−2.0 (IQR (−5.0, 0.0), *p* value = 0.002) with a significant rise in mindfulness (0.4 (IQR −0.1,0.8), *p* value = 0.03) between the baseline (T1) and the end of 4-week intervention (T2) measures. It is hard to ascertain the reason for this decline in the control group scores. It could be attributed to either “Placebo effect” as a result of participation in a research study or the positive effects of taking time off work and doing prescribed reading activity for the study duration.

During the cross-over period (from Week 4 to Week 8) when the control group received IEO training and the intervention group continued regular life as usual, there were no significant changes in any of the measured outcomes for the intervention group, except for a significant decline in Burnout subscale of Exhaustion (−1.5 (IQR −5.3, 1.0); *p* value = 0.04). We do not know whether the intervention group continued or discontinued the daily home practices and hence only speculate the reason for this decline in Exhaustion scores is due to an increased exposure to the IEO intervention. The control group, on the contrary, continued to experience a significant decline in anxiety scores (−1.0 (IQR −4.0,1.0); *p* value = 0.03) supporting our theory that workplace MBIs demonstrate moderate effects on “deficit-based” outcomes such as stress and anxiety [1, 8].

## 4. Discussion

The study seeks to contribute to the existing literature by introducing an emerging body-mind behavioral medicine intervention called “Inner Engineering Online (IEO).” The study evaluates the effect of IEO in alleviating dysfunctional experiences, such as burnout, stress, depression, and anxiety while promoting positive emotions among employees in an Information Technology company. We employed a randomized, active control group cross-over design. Though the overall results show that IEO did not have a significant effect on both negative and positive outcomes, there was a significant decline in perceived stress for the intervention group compared to the control group in the first phase in compliant participants. When the cross-over control group was subjected to IEO intervention, there was a significant decline in perceived stress compared to its own baseline. Below, we discuss plausible explanations of the findings.

First, similar to most studies of workplace MBIs, our study recruited participants through a company-wide awareness campaign that a well-being program was offered by the company. This open enrollment possibly resulted in a self-selection effect—the program offering attracted employees with relatively high baseline levels of negative states, such as burnout, stress, anxiety, and depression. The data show that baseline scores of these negative states were at the higher end for both the intervention and control groups. In contrast, the positive states such as mindfulness and joy were at the lower end of score range. In such cases, scores are usually subject to the effect of regression to the mean [8] for both the intervention and control groups. Our results show that both the intervention and control groups experienced significant amelioration of stress and significant improvement in mindfulness and joy, resulting in insignificant treatment effect.

Alternatively, burnout researchers may argue that there could be the “healthy worker's effect” [29], which is a well-documented selection bias in burnout research. It suggests that those who are ill, disabled, or have left the organization because of work-related stress are not included in the study sample. This might be the case for our study as the participants were recruited following an awareness campaign in the company helmed by the company's CEO. Since blinding was not incorporated, selection bias could be a limitation of our study.

Second, the active control group design, a more gender-balanced sample, and the small sample size and nonparametric tests may contribute to the insignificant treatment effect. The most common type of comparison group in MBIs and yoga studies is the usual care or passive waitlist control where no changes were made to the typical activities of the participants [14, 30], which may result in exaggerated treatment effect [15]. Although an active control is a more robust design, it is challenging to design a “placebo” active control activity [14, 15]. Treatment effect is harder to detect with active control groups. Furthermore, some researchers argue that using passive waitlist control may actually spuriously amplify the treatment effect between the intervention and the control because participants assigned to the passive waitlist control may expect to *not* get better without receiving the treatment [15] and recommend using an active control group for yoga research [14]. However, it is challenging to design an active control activity that resembles a “placebo” to the body-mind intervention [15].

The participants of workplace MBIs to date are predominantly women (about 73%) [6]. The samples of MBIs studies heavily skew toward the healthcare professionals or educators and are overwhelmingly women (about 73%) [6]. There is a dearth of studies investigating other fast growing professions, such as the Information Technology professionals. Although influential technological companies (e.g., Google and Intel) are among the pioneers in introducing MBPs at workplace [6], little evidence is available about the impact of these programs. The Bureau of Labor Statistics projects that employment in computer and information technology (IT) occupations will grow 11% from 2019 to 2029, much faster than the average growth of employment in all the other occupations together [31]. The IT professions are low in diversity—about 26% of them are women and 8% for African or for Latino Americans [32]. The lack of diversity in workplace MBIs calls for including participants who represent more diverse demographics and occupations [1]. Could a more gender-balanced sample result in the insignificant finding of treatment effect?

It is also desirable to diversify outcome measures of body and mind interventions beyond dysfunctional psychiatric issues (e.g., anxiety and depression). Positive outcomes such as emotional intelligence, positive emotions, vitality, flourishing, and life satisfaction and work-related outcomes such as job performance and work engagement await more research [1]. Unfortunately, the results show that IEO did not exercise a sustained significant effect on positive states, such as mindfulness and joy. This finding is similar to those of other MBIs that demonstrate small to no significant effect of workplace MBIs on emotional regulation and well-being [1, 8]. It is unclear why MBIs have small or inclusive effect on positive experiences. Fredrickson et al. [33] found that 7-week Loving-Kindness Meditation (LKM) training produced daily experiences of positive emotions, which in turn produced an increase in a wide range of personal resources, such as increase in mindfulness, purpose in life, and social support and a decrease in illness symptoms. Perhaps, positive emotions take longer time to develop. However, Zeng et al. [34] found that the length of LKM interventions and the time spent on meditation did not influence the effect sizes, but the studies without didactic components in interventions tend to have small effect sizes. Furthermore, a previous corporate pilot study found that IEO has a significant effect on vitality, joy, mindfulness, and work engagement [23]. Rangasamy et al. [35] found that learning and practicing a 15 minutes of Isha Kriya meditation (one of the introductory Inner Engineering methods) resulted in a reduction in mood disturbances such as tension, anger, fatigue, depression, and confusion. Further studies with a larger sample are needed to reexamine the effect of IEO on employee mental well-being.

Next, when compliance of daily practices is taken into account, IEO has a significant effect on reducing stress in the intervention group, compared to the active control group who engaged in reading a book during the 4-week intervention period. Similarly, IEO has a significant effect on reducing stress in the control group during their cross-over period, compared to their own baseline at the time cross over. The approach of body-mind behavioral medicine is to engage participants as stakeholders, who bear the responsibility of “doing the work” for their well-being. The caveat of this approach is that without doing the homework of daily practices, participants may not experience the intended effect of the intervention. Peterson et al. [24] found that adherence to Shambhavi Mahamudra Kriya (a 21-minute daily practice for which IEO is a prerequisite) resulted in decline in stress scores and improvement in well-being. Carmody and Baer [36] demonstrated a positive correlation between time spent in mindfulness practice and extent of improvement in measures of stress and well-being. In their systematic review on the role of home practice in mindfulness-based intervention, Lloyd et al. [37] found that four of the seven studies claimed that home practice predicted improvements in clinical outcome measures. These findings are consistent with our study's results that adhere to recommended home practices and have shown to play an important role in realizing the effect of the intervention on intended outcomes.

## 5. Conclusion

We attempt to contribute to the existing body-mind interventions in the workplace by introducing the novel IEO program and evaluating its effectiveness in reducing stress, burnout, anxiety, and depression and in inducing positive affect. Our study demonstrates that IEO has a significant effect on stress reduction only for participants who adhere to recommended daily yogic practices. These results point to the importance of participants' engagement in order to realize the benefits of mind-body interventions. Although our study has a rigorous experimental design by utilizing random assignment and active control, the study is limited by its small sample size. Future study may reexamine the effectiveness of IEO in promoting occupational health by using a larger and diverse sample.

## Figures and Tables

**Table 1:** Demographic and health characteristics of participants.

Characteristics	Intervention group (*n* = 35)		Control group (*n* = 35)	
	Mean	SD	Mean	SD
*Age*	40.5	(30–51)	38.7	(28.7–48.7)
				
	*n*	(%)	*n*	(%)

*Gender* ^*∗*^				
Male	16	(45.7)	18	(51.4)
Female	18	(51.4)	17	(48.6)

*Education*				
High school	0	(0.0)	1	(2.9)
Graduate	27	(77.1)	16	(45.7)
Postgraduate	8	(22.9)	18	(51.4)
Ph.D./doctorate				

*Health condition*				
Hypertension	3	(8.6)	5	(14.3)
Diabetes mellitus	1	(2.9)	2	(5.7)
Coronary artery disease	0	(0.0)	1	(2.9)

*Smoking*				
Active	2	(5.7)	0	(0.0)
Recent nonsmoke	1	(2.9)	0	(0.0)
Never	31	(88.6)	35	(100.0)

*Alcohol consumption*				
Social	19	(54.3)	24	(68.6)
Regular	4	(11.4)	0	(0.0)

*Prior experience of meditation*				
Yes	10	(26.8)	8	(22.9)

^*∗*^Missing data in intervention group.

**Table 2:** Mann–Whitney *U* test results between intervention group (*n* = 34) and control group (*n* = 34).

Mann–Whitney *U* test results between intervention group (*n* = 34) and control group (*n* = 35) (intergroup analysis at different time points for primary and secondary outcomes)													
Variables	Baseline (T1)–Week 4 (T2)				Week 4 (T2)–Week 8 (T3)			Week 8 (T3)–Week 12 (T4)				
	Intervention (*n* = 35)	Control group (*n* = 33)	Test stats (Mann–Whitney *U*)	*p* value	Intervention (*n* = 35)	Control group (*n* = 33)	Test stats (Mann–Whitney *U*)	*p* value	Intervention (*n* = 35)	Control group (*n* = 33)	Test stats (Mann–Whitney *U*)	*p* value
MBI-Exhaustion	−1.0 (−4.0; 2.0)	−1.0 (−3.0; 1.0)	597.5	0.81	−1.5 (−5.3; 1.0)	−1.0 (−3.3; 0.0)	558.5	0.89	−1.5 (−5.3; 1.0)	(−2.3; 2.0)	669.0	0.17
MBI-Cynicism	−0.3 (−0.9; 0.4)	−0.2 (−1.0; 0.3)	617.0	0.67	(3.3; 3.3)	−0.5 (−4.0; 3.0)	512.5	0.74	(−3.3; 3.3)	(−0.4; 3.0)	541.0	0.93
MBI-Professional Efficiency	0.5 (−2.0; 5.3)	0.0 (−2.0; 2.0)	530.5	0.55	(−3.0; 2.0)	(−5.3; 4.0)	593.0	0.66	(−3.0; 2.0)	(−3.0; 3.3)	586.5	0.73
PSS	−3.0 (−8.0; 0.0)	−1.5 (−4.0; 1.0)	703.0	0.19	−1.0 (−3.0; 2.0)	−2.5 (−6.0; 4.0)	488.0	0.27	−1.0 (−3.0; 2.0)	(−2.0; 3.0)	628.0	0.44
MAAS	0.3 (−0.1; 0.9)	0.4 (−0.1; 0.8)	587.5	0.93	(−0.4; 0.4)	0.1 (−0.2; 0.5)	675.0	0.28	(−0.4; 0.4)	(−0.2; 0.6)	697.0	0.17
DPES	0.3 (0.0; −0.8)	0.1 (−0.5; 0.7)	492.0	0.22	(−0.3; 0.8)	(−0.5; 0.7)	561.0	0.88	(−0.3; 0.8)	−0.2 (−0.8; 0.4)	469.0	0.17
CES-D	−1.0 (−5.0; 0.0)	−1.5 (−5.0; 3.0)	617.0	0.79	(−2.0; 4.0)	(−3.0; 2.0)	517.0	0.50	(−2.0; 4.0)	(−3.3; 2.3)	506.0	0.38
Anxiety	−3.0 (−7.0; 0.0)	−2.0 (−5.0; 0.0)	670.0	0.37	(−1.0; 3.0)	−0.5 (−4.0; 1.0)	405.5	0.03	(−1.0; 3.0)	(−1.0; 1.0)	544.0	0.72

**Table 3:** Wilcoxon signed-rank test for intragroup analysis between intervention group (*n* = 34) and control group (*n* = 34).

Wilcoxon signed-rank test results between intervention group (*n* = 34) and control group (*n* = 35) (intragroup analysis at different time points for primary and secondary outcomes)												
Variables	Intervention (*n* = 34)						Control group (*n* = 34)					
	T1–T2	*p* value	T2–T3	*p* value	T3–T4	*p* value	T1–T2	*p* value	T2–T3	*p* value	T3–T4	*p* value
MBI-Exhaustion	−1.0 (−4.0; 2.0)	0.11	−1.5 (−5.3; 1.0)	0.04	NA	NA	−1.0 (−3.0; 1.0)	0.09	−1.0 (−3.5; 0.0)	0.08	(−2.3; 2.0)	0.83
MBI-Cynicism	−0.3 (−0.9; 0.4)	0.13	0.0 (−0.7; 0.7)	0.79	NA	NA	−0.2 (−1.0;−0.3)	0.18	−0.2 (−0.8; 0.2)	0.40	0.0 (−0.4; 3.0)	0.59
MBI-Professional Efficacy	0.5 (−2.0; 5.3)	0.22	(−0.5; 0.3)	0.43	NA	NA	0.0 (−2.0; 2.0)	0.73	0.0 (−0.9; 0.7)	0.90	(−3.0; 3.3)	0.69
PSS	−3.0 (−8.0; 0.0)	0.01	−1.0 (−3.0; 2.0)	0.52	NA	NA	−1.5 (−4.0; 1.0)	0.04	−3.0 (−6.0; 4.0)	0.20	(−2.0; 3.0)	0.65
MAAS	0.3 (−0.1, 0.9)	0.01	(−0.4; 0.4)	0.70	NA	NA	0.4 (−0.1,0.8)	0.03	0.1 (0.2; 0.5)	0.13	(−0.2; 0.6)	0.08
DPES	0.3 (0.0;−0.8)	0.03	0.0 (−0.3; 0.8)	0.35	NA	NA	0.1 (−0.5,0.7)	0.40	0.0 (−0.6; 0.7)	0.66	−0.2 (−0.8; 0.4)	0.26
CES-D	−1.0 (−5.0, 0.0)	0.02	1.0 (−2.0; 4.0)	0.44	NA	NA	−1.5 (−5.0,3.0)	0.21	0.0 (−3.0; 2.0)	0.87	(−3.3; 2.3)	0.56
Anxiety	−3.0 (−7.0, 0.0)	0.001	0.0 (−1.0; 3.0)	0.30	NA	NA	−2.0 (−5.0,0.0)	0.002	−1.0 (−4.0; 1.0)	0.03	(−1.0; 1.0)	0.42

## Data Availability

The data used to support the findings of this study are available from the corresponding author upon request.
